# Effect of Embedded Pd Microstructures on the Flat-Band-Voltage Operation of Room Temperature ZnO-Based Liquid Petroleum Gas Sensors

**DOI:** 10.3390/s131216801

**Published:** 2013-12-05

**Authors:** Ghusoon M. Ali, Cody V. Thompson, Ali K. Jasim, Isam M. Abdulbaqi, James C. Moore

**Affiliations:** 1 Department of Chemistry and Physics, Coastal Carolina University, Conway, SC 29528, USA; E-Mail: cvthomps@g.coastal.edu; 2 Department of Electrical Engineering, Al-Mustansiriyah University, Baghdad 10052, Iraq; E-Mails: alijassim79@yahoo.com (A.K.J.); embaki56@yahoo.com (I.M.A.)

**Keywords:** zinc oxide, ZnO, interdigitated, liquid petroleum gas, LPG, sol-gel, metal-semiconductor-metal, gas sensor, palladium, Pd

## Abstract

Three methods were used to fabricate ZnO-based room temperature liquid petroleum gas (LPG) sensors having interdigitated metal-semiconductor-metal (MSM) structures. Specifically, devices with Pd Schottky contacts were fabricated with: (1) un-doped ZnO active layers; (2) Pd-doped ZnO active layers; and (3) un-doped ZnO layers on top of Pd microstructure arrays. All ZnO films were grown on *p*-type Si(111) substrates by the sol-gel method. For devices incorporating a microstructure array, Pd islands were first grown on the substrate by thermal evaporation using a 100 μm mesh shadow mask. We have estimated the sensitivity of the sensors for applied voltage from –5 to 5 V in air ambient, as well as with exposure to LPG in concentrations from 500 to 3,500 ppm at room temperature (300 K). The current-voltage characteristics were studied and parameters such as leakage current, barrier height, reach-through voltage, and flat-band voltage were extracted. We include contributions due to the barrier height dependence on the electric field and tunneling through the barrier for the studied MSM devices. The Pd-enhanced devices demonstrated a maximum gas response at flat-band voltages. The study also revealed that active layers consisting of Pd microstructure embedded ZnO films resulted in devices exhibiting greater gas-response as compared to those using Pd-doped ZnO thin films or un-doped active layers.

## 1. Introduction

Liquid petroleum gas (LPG) is a common fuel source used in industrial and domestic applications in all parts of the world. It is a highly flammable and potentially hazardous gas due to the potential for explosive combustion caused by undetected leaks. Due to the ubiquity of LPG as a fuel source, sensitive leak detection is necessary for a wide variety of applications. Specifically, the incorporation of sensitive LPG sensors in domestic and industrial appliances that utilize the gas could result in reliable, advanced safety feedback mechanisms [1–3].

Semiconductor metal-oxide LPG gas sensors have proven to be reliable and sensitive. Several different metal-oxide systems have been utilized as gas sensing materials, such as tin oxide (SnO_2_), tungsten trioxide (WO_3_), titanium oxide (TiO_2_) and zinc oxide (ZnO) [2–5]. ZnO has a unique combination of properties with respect to gas sensing. Specifically, ZnO is a non-toxic material with a wide direct band gap (3.37 eV at 300 K), high mobility of conduction electrons, good electrochemical and thermal stability under operating conditions, wide electrical conductivity range, and low fabrication cost [6]. It is inherently *n*-type because of the non-stoichiometry created by the presence of native donor defects, hydrogen defects, oxygen vacancies and/or zinc interstitials [2,5,7–10]. Therefore, it is not surprising that ZnO has been under intense investigation with respect to gas sensors, as well as other applications [3,5,11].

Gas sensors have been fabricated from various base ZnO forms, such as single crystals, sintered pellets, powder, thick films and thin films. Various device structures have also been used, such as heterojunction diodes, Schottky diodes, metal-semiconductor-metal (MSM) structure, thin film transistors (TFT), resistors, surface acoustic wave (SAW) and MOS capacitors [1–3,8,12–21]. Thin films and nanomaterials are suitable for gas sensors because the sensing properties are related to the material surface where the gases are adsorbed and surface reactions occur. Surface reactions change the concentration of charge carriers in the material, creating a depletion layer and surface dipole at the interface, which results in a change in electrical resistance [2–4,10,18,21–23]. The high sensitivity of ZnO thin film gas elements has been attributed to reactions at grain boundaries and the metal/ZnO interface, where the depletion of carriers modifies the material transport properties [2–4,18,21].

Although ZnO itself has an active gas response, its gas-sensing performance can be enhanced by the addition of a palladium (Pd) catalyst, which can be incorporated either via doping or embedding particles within the film [4,7,24–28]. Furthermore, Pd thin films have been studied as a Schottky barrier contact for ZnO-based gas sensors [12,29,30]. In the presence of the gas, a Schottky barrier is formed at the inter-grain boundaries of the film and Pd/ZnO interface, which dominates the conductivity of the film. Depending upon the type of gas and temperature, the Schottky barrier height changes, resulting in an increase or decrease in the conductivity. The sensing properties are found to depend on the temperature, grain size, catalyst and porosity [12]. Although there are numerous reports on the sensing properties of Schottky Pd/ZnO, there are few reports on the effect of palladium doping and embedding of Pd microparticles for this contact scheme [12,29,30].

In the present study we report on the fabrication and characterization of Pd/ZnO interdigitated MSM LPG sensors having three different arrangements. Specifically, devices with Pd Schottky contacts were fabricated with (1) un-doped ZnO active layers; (2) Pd-doped ZnO active layers; and (3) un-doped ZnO layers on top of Pd microstructure arrays. The electrical characteristics and gas response of the devices were studied and compared to explore the potential applications of these configurations as room temperature LPG sensors.

## 2. Experiment

**Figure 1.**
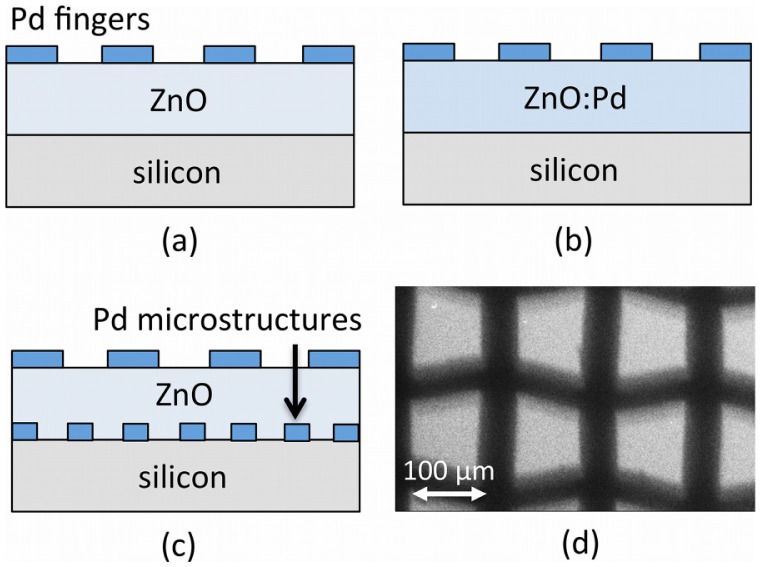
Schematic diagram of the metal-semiconductor-metal (MSM) gas sensor geometries for devices based on (**a**) un-doped zinc oxide (ZnO) films; (**b**) Pd-doped ZnO films; and (**c**) ZnO films deposited on Pd microstructures; (**d**) SEM image of a metal microstructure array thermally evaporated through a 100 μm aperture sieve (scale bar=100μm).

Figure 1 shows a schematic diagram of the MSM photodetector geometry for the three device structures under study: (a) un-doped ZnO active layers; (b) Pd-doped ZnO; and (c) un-doped ZnO active layers on Pd microstructure arrays. We deposited ZnO thin films using the sol-gel technique, and we fabricated MSM device contacts and microstructure arrays by thermal evaporation of Pd using a shadow mask technique.

The substrates used for deposition were *p*-type Si(111) (∼380 μm thick) with a resistivity of 2–7 Ωcm. Before deposition of the ZnO films, the substrates were ultrasonically agitated in trichloroethylene for 5 min, immersed in acetone for 1 min, and cleaned in a solution consisting of 40% H_2_SO_4_ and 60% H_2_O_2_. To remove the native silicon oxide layer, substrates were dipped in a 6:1 solution of deionized water and HF and then quenched in a beaker containing deionized water [11].

Zinc acetate dihydrate is well known as a starting material for the preparation of ZnO sols for coatings. It is a low cost material that has a good solubility as compared to alkoxides like zinc-n-propoxide in alcohols. However, zinc acetate dihydrate has only limited solubility in alcohols in the absence of other agents or heating. For un-doped solution, 2.19 g of zinc acetate dehydrate (Zn(CH_3_COO)_2_·2H_2_O:ZnAc_2_·2H_2_O) was dissolved in a solution consisting of 20 mL isopropanol ((CH_3_)_2_CHOH) and 0.8 g diethanolamine (DEA: [CH_2_(OH)CH_2_]_2_NH) at room temperature (300 K). The DEA to zinc acetate molar ratio was kept to 1:1 [11]. For Pd-doped solution, 0.23 g of Palladium (II) nitrate (Pd(NO_3_)_2_, Merck) was dissolved in a solution of isopropanol and the desired amount of DEA. The concentrations were 0.5 mol/L for zinc acetate, and 0.05 mol/L for Palladium (II) nitrate. The molar ratio of Pd as a dopant was 10 at.% with respect to Zn. The resultant solutions were stirred at room temperature for 1 h to yield a clear and homogeneous solution that was used for coating [31].

For un-doped ZnO films deposited on Pd microstructures, metal arrays were fabricated with the help of a shadow mask technique. A standard sieve mesh with aperture width of ∼100 μm was used as a mask during the thermal evaporation of Pd powder (99.99%) on cleaned Si substrates. This resulted in a uniform array of Pd microstructures having widths of approximately ∼100 μm, as shown in the SEM image in Figure lc. ZnO films were prepared by spin coating the sol-gel on the wafers for 40 s at a speed of 4,000 rpm. This step was followed by preheating the coating at 373 K for 10 min and post-heating at 723 K for 1 h in an air-ambient muffle furnace.

Arrays of four planer type MSM diodes were created on all samples (50.8 mm diameter Si substrates). MSM sensors were fabricated on the top surface of the ZnO films with an interdigited finger electrode, as shown schematically in Figure la–c. Pd was deposited via thermal evaporation using a shadow mask fabricated by a wire cut machine (model W-A430, ACRA, USA). The deposited fingers were designed to have a width of 150 μm, a length of 4,000 μm, and a spacing of 150 μm. Immediately prior to the fabrication of metal electrodes, the wafer was dipped in an organic solution and deionized water.

Surface morphology of the ZnO films was studied by atomic force microscopy (AFM). The surface properties of the samples, such as the surface roughness and grain size, were measured by AFM (Angstrom Advance AA3000, tip NSC35/AIBS). The crystal structures of the ZnO films were characterized by X-ray diffraction (XRD) using Cu-K*α*; radiation in the range 2*θ* from 20° to 80°. For the investigation of the carrier concentration and mobility, Hall effect measurements were performed using an Ecopia HMS 3000. The thicknesses of the Pd-doped and Pd microparticle embedded ZnO films were estimated to be between 250 and 300 nm, as measured by TFProbe from Angstrom Advance Inc.

The current-voltage characteristics were measured using a semiconductor characterization system (SCS-4200, Keithley) at room temperature (300 K) for applied voltage ranging between –5 to +5 V. The fabricated MSM LPG gas sensors were studied in the air as well as during exposure to LPG in concentrations of 500 to 3,500 ppm at room temperature (300 K).

## 3. Characterization of Active Layers

**Figure 2.**
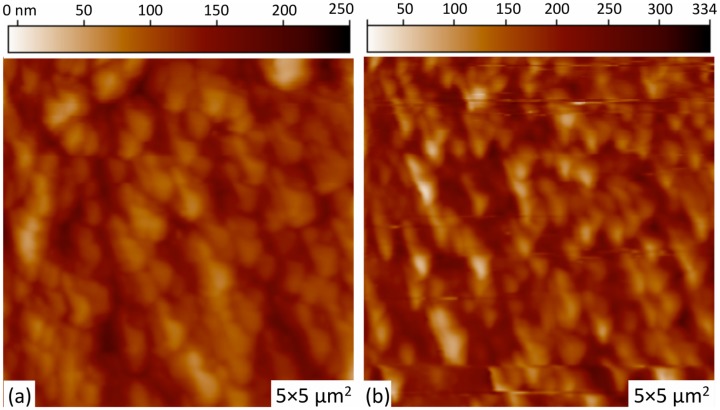
Atomic force microscopy (AFM) topography images of (**a**) the Pd-doped ZnO film surface, and (**b**) the surface of the un-doped ZnO film grown on top of a Pd microstructure array. Both images are 5 × 5 μm^2^, with the greyscale showing Δz of 250 nm and 330 nm for (**a**) and (**b**), respectively.

The influence on the ZnO surface morphology due to Pd incorporation schemes is shown in Figure 2a,b. All surfaces showed good homogeneity with no surface cracks. Both ZnO films are seen to be polycrystalline in nature, with no significant difference in particle grain size between the two Pd-doping schemes, which is confirmed by the XRD patterns discussed below. Figure 2a,b shows the formation of vertically well-aligned ZnO grain arrays, with greater array ordering and homogeneity observed for the un-doped ZnO film on Pd microstructures. Un-doped ZnO films without Pd microstructures demonstrate similar morphology to that shown in Figure 2b. The root mean square (rms) value of roughness for the Pd-doped ZnO and un-doped ZnO on Pd microstructures are measured to be 39 nm and 58 nm, respectively. The average grain sizes were measured to be 207 nm and 222 nm, respectively. The incorporation of large metal islands on the substrate does not appear to have an effect on the morphology of the subsequently grown sol gel films. However, the size of the microstructures in this study are several orders of magnitude larger than the morphological features of the active layer film.

**Figure 3.**
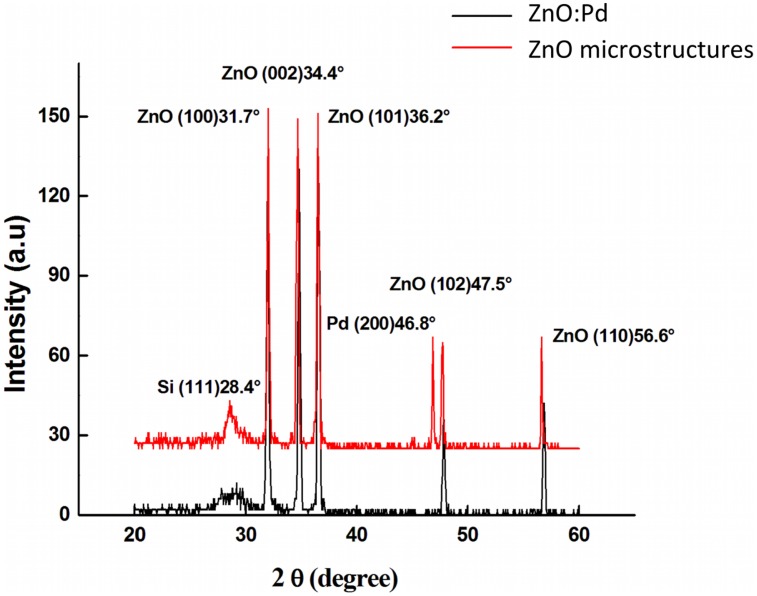
XRD pattern of the ZnO:Pd and Pd microstructure embedded ZnO films grown by sol-gel technique on Si substrate.

Figure 3 shows the XRD pattern for both the Pd-doped ZnO and the Pd microstructure embedded films. The diffraction peaks for all films indicate that they possess a polycrystalline hexagonal wurtzite crystal structure [32]. Furthermore, the small line widths of all peaks indicate that the films are well oriented with the pure wurtzite structure. Specifically, for Pd-doped ZnO thin films six peaks appear at 2*θ* from 20° to 70° that correspond to the (001), (002), (101), (102), and (110) directions of the hexagonal ZnO crystal structure. There is no dominant peak. One other peak can be observed at 28.4° that corresponds to the (111) orientation of silicon. No peaks corresponding to the dopant are observed, which may be due to the small size of Pd particles and the low amount of Pd surface species. Nearly identical patterns are observed for Pd microstructure embedded films. However, an additional peak is observed at 46.8° that corresponds to the (200) orientation of Pd [33]. The electronic parameters were measured by van der Pauw and Hall methods, and are summarized in Table 1. Surface resistivity *ρ* is found to be 3.49 and 3.04 × 10^2^ Ωcm for the Pd-doped and Pd microstructure films, respectively. Hall mobility μ was 45.82 and 34.68 cm^2^V^−1^s^−1^, and carrier concentration was 3.84 × 10^16^ and 6.10 × 10^14^ cm^−3^, respectively. The resistivity decreases by two orders of magnitude due to doping, which can be attributed to the increase in the carrier concentration and mobility. Mobility augmentation is typically attributed to an increase in the grain size with doping. Charge carriers are scattered at grain boundaries, resulting in lower mobility with increased grain boundary density Therefore, when the grain boundary density decreases the mobility increases. This should result in a decrease in the surface resistivity with increasing grain size. However, there is no significant difference in grain size observed between the two films. Beck *et al.* attribute this to the creation of a depletion layer at the interface between the metal microstructure arrays and the *n*-type ZnO, which creates a smaller effective grain size with respect to charge carriers [34]. The increase in the resistivity due to microstructure incorporation can be attributed to this alignment of the Fermi level of the Pd with that of ZnO in the thin films.

## 4. Current-Voltage Characterization of Devices

**Figure 4.**
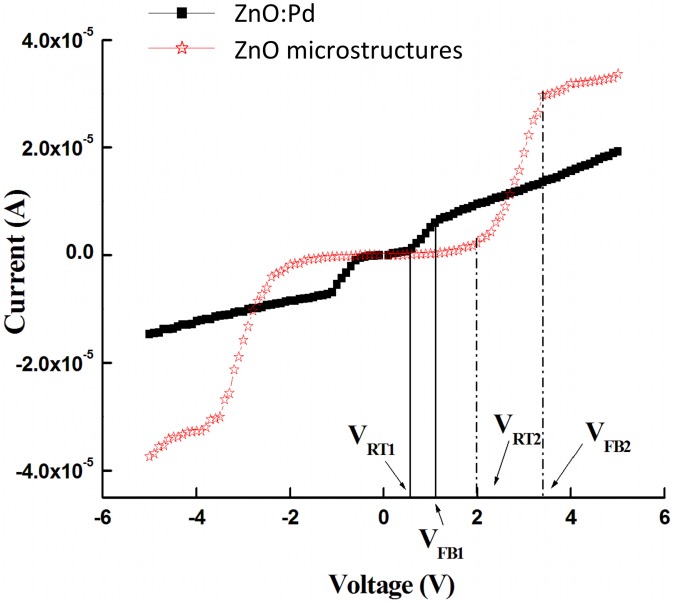
Room-temperature current-voltage response for Pd MSM device structures based on Pd-doped (filled squares) and Pd microstructure embedded (empty stars) active layers.

Devices based on un-doped ZnO active layers demonstrated no LPG response, as we will address later. Therefore, we will primarily discuss the Pd-enhanced devices in the following section. Figure 4 shows the current-voltage characteristics measured in air-ambient at room temperature (300 K) for the MSM structures based on Pd-doped (filled squares) and Pd microstructure embedded (empty stars) active layers. The shape of the curves indicates the existence of Schottky barriers on the anode and cathode sides of the detectors. The figure shows three regions of operation in each bias direction: applied biases (1) before the reach-through voltage *V_RT_*; (2) between *V_RT_* and the flat-band voltage *V_FB_*; and (3) past *V_FB_*. These three regions are discussed in the following paragraphs.

At small voltages only small currents flow since one contact is in reverse bias. The hole current is much smaller than the electron current. Only those holes that diffuse through the neutral region contribute to the hole current. With increasing applied bias, the depletion region near the cathode grows and the depletion region at the anode shrinks. Eventually, the cathode depletion region reaches through to the anode and the semiconductor is entirely depleted at reach-through voltage *V_RT_*. The linear slope of the current-voltage curve for the region before *V_RT_* indicates a very high specific resistivity [35,36].

As the voltage increases past *V_RT_*, the linear slope changes. A point is reached for which the field in the semiconductor becomes unidirectional between cathode and anode at the flat-band voltage *V_FB_*. When the absolute voltage is between *V_RT_* and *V_FB_*, the current-voltage relationship is approximately linear, but with a slope several times larger. This behavior is attributed to a reduction in the hole injection barrier after reach-through, leading to stronger hole injection. Beyond the flat-band voltage the hole current increases only weakly since a lowering of the barrier occurs only via the Schottky effect [36]. In other words, the two voltages that demarcate the three regimes are (a) reach-through voltage *V_RT_*, which separates the full depletion regime from the depleted; and (b) the flat-band voltage *V_FB_*, which demarcates the full depletion regime [36].

The flat-band and reach-through voltages for both detector structures, as well as other device performance ratings, are detailed in Table 2 (obtained via inspection of Figure 4). Figure 4 shows that the reach-through voltages for devices based on Pd-doped and Pd microstructure embedded films are 0.56 V and 2.0 V, respectively. Flat-band voltages are 1.1 V and 3.3 V, respectively. The current-voltage measurements are in good agreement with the Hall-effect measurements. The difference in *V_RT_* and *V_FB_* between the two device structures can be attributed to the difference in the carrier concentration, where higher carrier concentration results in a narrower depletion layer that in turn leads to lower *V_RT_* and *V_FB_*.

The values of barrier height *ϕ_B_*, ideality factor *η*, and saturation current *I_s_* can also be extracted from the current-voltage characteristics and thermionic emission theory. A MSM sensor is a unipolar device with two back-to-back Schottky junctions formed on the same semiconductor surface. The current across a Schottky diode is governed by thermionic emission theory and is given by the following:

(1) $$I=AA^{*}T^{2}\exp\left(-\frac{q\phi_{B}}{kT}\right)\;\left[\exp\left(\frac{qV}{\eta kT}\right)-1\right]$$

where *A* is the Schottky contact area, A* is the effective Richardson constant (theoretically A* = 32 × 10^4^ Am^2^K^2^ for ZnO using m* = 0.27 m*_o_*), *T* is the absolute temperature, *q* is the charge of an electron, and *k* is the Boltzmann constant [35,37].

Past the flat-band voltage, Figure 4 shows slowly increasing current with applied bias for both devices and no indication of current saturation, which is in good agreement with previous reports [38,39]. The increasing saturation current for a Schottky contact with applied reverse bias can be explained in terms of the barrier height dependence on the electric field strength in the barrier as a result of the existence of an interfacial layer between the metal and the semiconductor.

The lack of saturation can also be caused by tunneling effects and carrier recombination. The tunnel current component of the leakage current increases abruptly as the voltage drops across the barrier reducing the effective barrier height. The tunnel current depends exponentially on the barrier height and width, which results in the rising current with applied bias [38]. As a result, the current-voltage characteristics of the Schottky contact are more appropriately analyzed in the more general form of equation, as follows:

(2) $$I=AA^{*}T^{2}\exp\left(-\frac{q\phi_{B}}{kT}\right)\exp\left(\frac{qV}{\eta kT}\right)\;\left[1-\exp\left(-\frac{qV}{kT}\right)\right]$$

Equation (2) includes barrier height lowering mechanisms, such as electric field, tunneling effects, and carrier recombination in the space-charge region of the metal-semiconductor contact. Note that when *V* > 3*kT*/*e* there is no difference between Equations (1) and (2) [38,39] . Within the constraints of this study, Equation (2) can be simplified to the following:

(3) $$\ln\;\left[I\exp\left(\frac{qV}{kT}\right)\right]=\ln I_{s}+\left(\frac{qV}{\eta kT}\right)$$

plotting the left-hand-side of Equation (3) as a function of applied bias *V* should give a straight line with y-intercept at ln(*I_s_*) and slope *q*/(*ηkT*), which allows the extraction of the saturation current and ideality factor. The intercepts for the devices under study result in saturation currents *I_s_* of 5.9 × 10^−6^ A and 1.4 × 10^−5^ A for the Pd-doped and microstructure embedded devices, respectively, as shown in Table 3.

The calculated value of the ideality factor for both devices show departure from unity, which suggests that the current transport cannot be described by thermionic transport alone. The tunneling transport mechanisms and existence of surface states must be taken into account for this purpose [11]. The ZnO thin films used in this study are interfacial layers with a thickness of 200 to 300 nm (including adsorbed oxygen, hydrogen, hydroxide, *etc.*) on the surface. As a result there exists abundant surface states on the ZnO surface [40–42]. The surface states form a potential barrier on the surface, which is almost independent of the work function of the metal due to Fermi level pinning [10,40].

When the density of surface states is high enough, the barrier height is controlled predominantly by the surface states leading to non-ideal diode characteristics with a high value of ideality factor [10,11]. The values of barrier height *ϕ_B_* at room temperature (300 K) were found to be 0.58 and 0.56 for the Pd-doped and microparticle embedded devices, respectively.

## 5. Results and Discussion for Liquid Petroleum Gas Sensors

**Figure 5.**
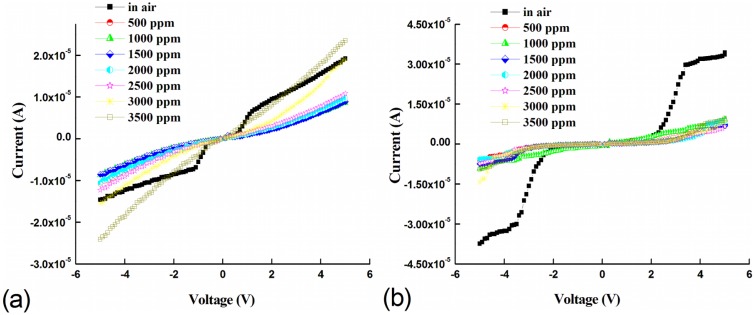
Room temperature current-voltage response for Pd MSM device structures based on (**a**) Pd-doped and (**b**) Pd microstructure embedded active layers. The liquefied petroleum gas (LPG) concentration is varied from 500 to 3,500 ppm.

Figure 5a,b shows the room temperature (300 K) current-voltage response for the Pd-enhanced devices at various LPG concentrations. Both devices show a LPG concentration dependent response under bidirectional voltage operation at room temperature. Interestingly, the Pd MSM devices with un-doped active layers and no microstructure embedding show no LPG gas response within the concentration range studied (not shown). For Pd-enhanced devices, the strong rectifying behavior observed in air-ambient for both devices transitions to quasi-ohmic behavior with the introduction of LPG within the range of concentrations studied. At flat-band voltages, a decrease in the current is observed as the LPG concentration is increased. The decrease in current at room temperature can be attributed to gas desorption and adsorption at the surface and dipole creation at the Pd/ZnO interface [21,43]. In air-ambient, oxygen is adsorbed by capturing a free electron from the surface creating a depletion layer that reduces film conductivity compared to vacuum [10,44]. Similarly, adsorption of LPG constituent molecules at the interface can further reduce conductivity by acting as trap states for conduction electrons [5,17,45–47]. Specifically for ZnO, the surface assists this continued adsorption of gases due to low surface atomic coordination and high surface energy [40].

The reversion from rectifying to quasi-ohmic behavior can be attributed to the catalytic decomposition of LPG (CH_4_, C_3_H_8_, C_5_H_10_, *etc.*) on the Pd metallization, followed by diffusion to the underlying ZnO interface. This diffusion results in an interfacial dipole layer at the Pd/ZnO interface that can decrease the Schottky barrier, resulting in quasi-ohmic behavior for the Pd contacts [21,43]. For room temperature gas sensors, this potential barrier dependent behavior dominates the gas response, where adsorption of gas species and dipole layer creation are the dominant mechanisms. However, at higher temperatures the thermal energy is sufficient to react with ambient O_2_ species, resulting in significant chemisorption [48].

**Figure 6.**
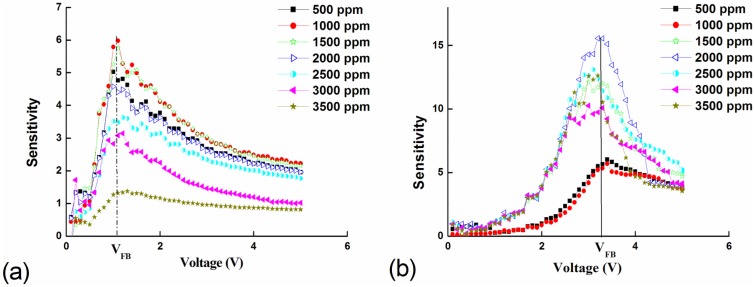
Sensitivity as a function of applied bias for Pd MSM device structures based on (**a**) Pd-doped and (**b**) Pd microstructure embedded active layers. The LPG concentration is varied from 500 to 3,500 ppm.

The sensitivity *S* was calculated using the following relationship:

(4) $$S=\frac{I_{\text{air}}}{I_{\text{LPG}}}$$

where *I_air_* and *I_LPG_* are the device currents in air and during exposure to LPG, respectively. Figure 6a,b shows the sensitivity as a function of applied bias for both devices at various LPG concentrations. For both devices, maximum device sensitivity across all concentrations is achieved at the flat-band voltage *V_FB_*. For devices based on Pd-doped active layers, the sensitivity remains relatively constant with increasing LPG concentration up to 2,000 ppm and then dramatically decreases with increasing concentration, as shown in Figure 6a. In contrast, for devices based on Pd microstructure embedded films, the sensitivity at *V_FB_* increases significantly with increasing LPG concentration up to 2,000 ppm, and then slowly decreases, as shown in Figure 6b.

**Figure 7.**
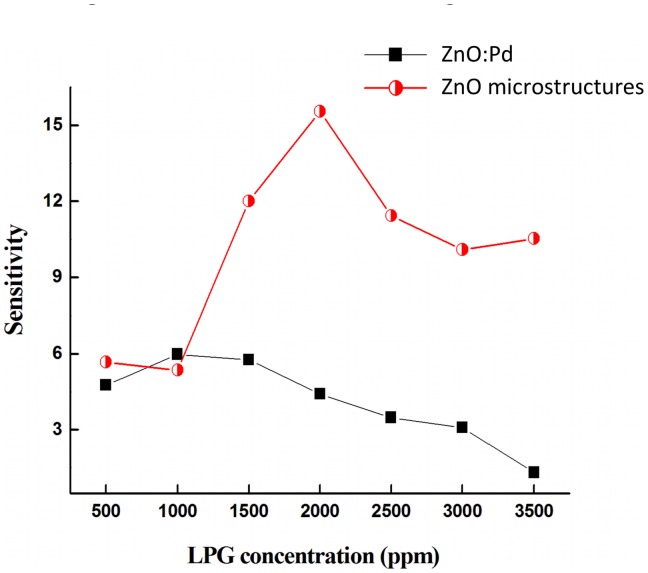
Maximum sensitivity at *V_FB_* as a function of LPG concentration. Filled squares show sensitivity for devices having Pd-doped active layers, and half-filled circles show sensitivity for devices having microstructure embedding.

Figure 7 shows the maximum sensitivity at *V_FB_* for both devices as a function of LPG concentration. The maximum sensitivity *S_max_* is observed at *V_FB_* equal to 1.1 V and 3.3 V for devices based on Pd-doped films and Pd microstructure embedded films, respectively. There is no significant difference in *S_max_* at low LPG gas concentrations (500–1,000 ppm). However, with increasing LPG concentration the devices based on Pd microstructure embedded films show a significant improvement in sensitivity.

The maximum sensitivity *S_max_* for both devices at *V_FB_* is shown in Table 3. It is clearly seen that sensors based on Pd microstructure embedded films (half-filled circles) have better sensing capability at higher voltage compared with Pd-doped devices (filled squares). Furthermore, Pd MSM devices with un-doped active layers and no microparticle embedding show no LPG sensitivity within the concentration range studied (not shown), suggesting that the sensing mechanism is primarily due to the incorporation of the Pd catalyst in the active layer. The enhancement in the sensitivity with large Pd embedding *vs.* small particle doping may be attributed to the creation of isolated depletion layers at the Pd microstructure/ZnO interface. These depletion layers enhance the resistivity, which decreases the current before the reach-through voltage, as seen in Figure 4. With increasing bias past reach-through, the current significantly increases as compared to Pd-doped films. The incorporation of larger underlying Pd/ZnO interfacial surface area appears to dramatically affect the *V_RT_* to *V_FB_* operation region, which results in improved sensitivity with respect to gas sensing.

## 6. Summary

In summary, we have reported on the fabrication and characterization of Pd/ZnO interdigitated MSM LPG sensors. Specifically, the sensing properties of un-doped, Pd-doped and Pd microstructure embedded ZnO active layers were compared. The current-voltage characteristics of the Schottky contact were examined by taking into account lowering of the barrier height in reverse bias. Devices based on Pd-doped films exhibited lower reach-through and flat-band voltages as compared to microstructure embedded devices, which was attributed to the existence of depletion regions at the micro-metal/semiconductor interface. The gas-response properties under different concentrations of LPG were studied at room temperature. It was found that there is no significant difference between Pd-doped and microstructure embedded devices at low gas concentrations (500–1,000 ppm). However, the performance of the microstructure devices was significantly improved at higher LPG concentration. Furthermore, the studied gas sensors operate most effectively at a flat-band bias condition. The improvement of device performance due to micro-metal embedding can be attributed to the combined effects of the depletion layer and surface dipole creation at the Pd/ZnO interface. Devices based on the embedding of micro-metal arrays could find applications in room temperature gas sensing devices.

## Figures and Tables

**Table 1.** Thin film comparative characteristics for the active layer.

***Parameter***	**Pd-Doped**	**Pd Microstructures**
roughness (nm)	39	58
grain size (nm)	207	222
film thickness (nm)	250	300
*ρ* (Ωcm)	3.49	304
μ (cm^2^ V^−1^ s^−1^)	45.82	34.68
carrier concentration (cm^−3^)	3.84 × 10^16^	6.10 × 10^14^

**Table 2.** Air-ambient, room temperature device characteristics of the MSM sensors.

***Parameter***	**Pd-Doped**	**Pd Microstructures**
*V_RT_* (V)	0.56	2.0
*V_FB_* (V)	1.1	3.3
*I_s_* (mA)	5.9 × 10^−3^	1. 4 × 10^−2^
*ϕ_B_* (eV)	0.58	0.56

**Table 3.** Performance parameters for LPG sensors.

***Parameter***	**Pd-Doped Pd Microstructures**	
*S_max_* @ *V_FB_* (V)	5.9	15.5
LPG concentration @ *S_max_* (ppm)	1000	2000
